# The Influence of Deterioration of Kidney Function on the Diagnostic Power of Laboratory Parameters Used in the Prognostic Classification of AL Amyloidosis

**DOI:** 10.3390/jcm10214903

**Published:** 2021-10-24

**Authors:** Emilia Czyżewska, Olga Ciepiela

**Affiliations:** Department of Laboratory Medicine, Medical University of Warsaw, Banacha 1a, 02-097 Warsaw, Poland; emilia.czyzewska@wum.edu.pl

**Keywords:** primary AL amyloidosis, eGFR, Kumar’s classification, immunoglobulin free light chains

## Abstract

There is a possibility that renal dysfunction may potentially reduce the diagnostic power of the laboratory parameters Tn, NT-proBNP and sFLC levels, used in the current prognostic classification of AL amyloidosis and the diagnosis of heart involvement by amyloid. In this study, the impact of lowering the eGFR value on the usefulness of these parameters in the prognosis and diagnosis of the presence of amyloid in the myocardium was assessed in a group of 71 patients with newly diagnosed primary amyloidosis. The assessment of diagnostic power of laboratory parameters was performed on the entire study group, and in the ranges of eGFR ≥ 60 and < 60 mL/min/1.73 m^2^. It has been proven that, with a decrease in the eGFR value, the concentrations of NT-proBNP and the κ uninvolved light chains increase significantly (*p* < 0.001). To assess the diagnostic power of laboratory parameters used in the diagnosis of myocardial involvement in patients with AL amyloidosis, an ROC analysis was performed. The highest values of AUC were obtained for the NT-proBNP concentration (AUC = 0.906). The lowest values of the AUC and Youden’s index were obtained for the dFLC values (AUC = 0.723), and involved κ FLC concentration (AUC = 0.613). For all compared parameters, the smallest values of the AUC were obtained for eGFR (<60 mL/min/1.73 m^2^). It seems that the most suitable cardiac parameter used in the prognostic classification of AL amyloidosis, independent of renal function, is TnI. It should be noted that a concentration of involved λ chains hada higher diagnostic power to assess the heart involvement, compared to the routinely used “cardiac parameters”, TnI and NT-proBNP. It can therefore be an additional parameter used to assess the presence of amyloid in the myocardium. A decrease in eGFR value influenced the change in the diagnostic cut-off points of the most analyzed laboratory parameters. Finally, it is concluded that lowering the eGFR value reduces the utility of laboratory parameters used in the prognostic classification of AL amyloidosis.

## 1. Introduction

Immunoglobulin light chain amyloidosis (AL) is a neoplastic disease in which the amyloid fibers are made of κ or λ immunoglobulin free light chains (FLC), or its fragments, produced by a clone of plasmacytes or lymphoplasmacytes. The produced protein adopts a β-sheet structure and, by being deposited extracellularly in tissues and organs, leads to impairment of their functions [1,2,3]. Cardiac involvement is a major adverse prognostic factor in AL amyloidosis, since 75% of deaths in this group of patients are caused by heart failure or arrhythmias due to amyloid fibril deposition. Cardiomyopathies are the main cause of death in patients with amyloidosis. Diagnosis of cardiac involvement is late, owing to non-specific symptoms and no early characteristic changes observed in imaging studies. The median survival of patients with no effective treatment is only ca. 4–6 months. The currently applicable prognostic classification of AL amyloidosis according to Kumar et al. is based on biochemical parameters assessing the myocardial function and damage degree (the concentration of troponin (Tn) and the N-terminal fragment of the B-type natriuretic peptide (NT-proBNP)), and the difference between the concentration of FLCs involved and uninvolved in the neoplastic process (dFLC) value [4,5,6]. Of importance to diagnostics may not only be the concentration of κ and λ free light chains, but also the ratio of their concentrations (κ/λ), being a somewhat numerical index of clonality in the production of one of the chains by pathological plasmacytes. Furthermore, it is noted that the serum concentrations of both Tn, NT-proBNP and FLC also depend on renal function, expressed as an eGFR value. The currently applicable prognostic classification does not consider the different cut-off points of concentrations of laboratory parameters in patients with normal and impaired renal function. There is a possibility that renal dysfunction may potentially reduce the diagnostic power used to assess amyloid deposits in the heart [7,8,9,10,11,12].

The goal of the study was to analyze the utility of laboratory parameters used in prognostic classification and assessment of myocardial involvement in patients with AL amyloidosis, with the consideration of the influence of lowering the eGFR value on their diagnostic power.

## 2. Materials and Methods

### 2.1. Study Group

The study included 71 patients (34 women and 37 men) of the Department of Hematology, Oncology, and Internal Diseases, Medical University of Warsaw, with primary AL amyloidosis according to the current criteria of diagnosis and organ involvement [2,13,14,15]. The criteria for the diagnosis of primary systemic AL amyloidosis included: 1. presence of secondary organ abnormalities resulting from amyloid deposition (involvement of the heart, kidneys, liver, gastrointestinal tract, and peripheral nervous system); 2. the presence of amyloid confirmed by Congo red staining of tissue biopsies (organ biopsy, adipose tissue, bone marrow, gingiva); 3. confirmation that amyloid is made of monoclonal immunoglobulin light chains (immunohistochemistry, electron microscopy, MS); 4. confirmation of the presence of plasma cell dyscrasias (presence of monoclonal protein in the serum and/or urine, an incorrect value of the ratio of the concentrations of free light chains in the serum, κ and λ in the serum, clonality of plasmacytes in the bone marrow). All four of the above criteria had to be met for the diagnosis of primary systemic AL amyloidosis. In localized amyloidosis, the only diagnostic criterion was the presence of amyloid localized in one organ, composed of light chains of immunoglobulins, without the presence of monoclonal protein in serum and/or urine. In this group of patients, a retrospective analysis of the results obtained in the period January 2009–May 2016 as part of the initial diagnosis (before treatment was applied) was performed [13,14,15]. The mean age at diagnosis was 58 ± 11 years. A total of 57 (80%) of the cases were patients with primary systemic light chain amyloidosis, 4 (6%) had localized primary light chain amyloidosis, and 10 (14%) patients had primary systemic light chain amyloidosis associated with myeloma. In 43 (61%) patients, the amyloid was made of λ chains, and, in 28 (39%), κ chains. In 22 (31%) patients, the amyloid was in one organ, in 28 (40%), in two organs, and in 18 (25%), in three or more. In three patients (4%), the amyloid was found only in the bone marrow or in the adipose tissue. In 51 (72%) patients, which constitute a study group, the organ involvement by amyloid involved the heart, in 41 (58%), the kidneys, in 10 (14%), the liver, in 20 (28%), the gastrointestinal tract, and in 14 (20%), other (bronchi, lungs, peripheral nervous system, nasopharynx, tongue, skin). No involvement of the heart was diagnosed in 20 of included subjects (28%), and these subjects constitute control group. Exclusion criteria were, among others: non-amyloid heart disease; history of hypertension; valvular disease and atrial fibrillation; hyperthyroidism; primary hyperaldosteronism; acute kidney damage caused by Cushing’s syndrome; cirrhosis of the liver with ascites; subarachnoid hemorrhage; stroke; injuries of the central nervous system; intake of thyroid hormones; sepsis; and obesity. These data were obtained with the consent of clinicians from medical records. 

Comparative analysis of all included parameters was performed in the entire study group, depending on the type of monoclonal chain forming amyloid, and in groups depending on the severity of chronic kidney disease (CKD) according to Kidney Disease: Improving Global Outcomes (KDIGO) CKD Working Group: KDIGO 2012 [16], based on the eGFR values according to CKD-EPI: stage 1 group (G1) eGFR ≥ 90 mL/min/1.73 m^2^ and stage 2 group (G2) eGFR: 60–89 mL/min/1.73 m^2^ (A); stage 3 group (G3a and G3b) eGFR: 30–59 mL/min/1.73 m^2^ and stage 4 group (G4) eGFR: 15–29 mL/min/1.73 m^2^ (B); stage 5 group (G5) eGFR: <15 mL/min/1.73 m^2^ (C). The analysis of the correlation between the concentration of κ and λ free light chains (involved and uninvolved), the dFLC value, and the κ/λ ratio with the biochemical parameters of heart involvement and eGFR values was performed in the group of patients with myocardial involvement, depending on the eGFR value (eGFR ≥ and < 60 mL/min/1.73 m^2^). The assessment of diagnostic power (diagnostic sensitivity, diagnostic specificity, analysis of ROC curves, and AUC values) was carried out in the entire study group, and in the ranges of eGFR ≥60 and <60 mL/min/1.73 m^2^. In the comparative analysis, the reference group were patients diagnosed with primary AL amyloidosis without amyloid deposits in the myocardium.

### 2.2. Laboratory Methods

The ĸ and λ FLC concentration was measured through the immunoturbidimetric method on Cobas c501 (Roche, Rotkreuz, Switzerland), using Freelite^®^ Human Kappa/Lambda (Binding Site, Birmingham, UK). The reference range was κ: 3.70–19.40 mg/L, and λ: 5.71–26.30 mg/L. Subsequently, the ratio of ĸ/λ FLC in the serum (reference range 0.26–1.65) and dFLC were calculated. NT-proBNP and TnI concentrations were measured using a Dimension EXL analyzer (Siemens Healthcare Diagnostics, Newark, NJ, USA), with the use of a one-step sandwich chemiluminescent method, based on the Luminescent Oxygen Channeling Immunoassay (LOCI^®^) method. The reference values range regarding NT-proBNP were less than 125 pg/mL for patients aged 75 and younger, and less than 450 pg/mL for patients older than 75 years old. The reference values range for TnI were 0.000–0.056 ng/mL. The eGFR value was calculated according to the CKD-EPI formula, based on the determination of the creatinine concentration, using the standardized method (compensated Jaffe reaction, on Cobas c501, Roche, Rotkreuz, Switzerland) [17]. Reference values range: >60 mL/min/1.73 m^2^.

### 2.3. Statistical Analysis

The results are presented as median and an interquartile range (IQR), or absolute values and percentages where applicable. The Kolmogorov–Smirnov test was used to assess the normality of the distributions. In the comparative analysis of the tested material, depending on the type of amyloid forming monoclonal chain (κ,λ), the Wilcoxon test was performed. In the comparative analysis of the tested material, depending on the eGFR values, the ANOVA on ranks test was implemented. Correlation analysis was performed using the Spearman test. Bonferroni correction was used to counteract the problem of multiple comparisons. To assess the diagnostic power of laboratory parameters used in the diagnosis of myocardial involvement in patients with AL amyloidosis, the following values were calculated: sensitivity, and specificity, as well as positive and negative predictive values. The receiver-operating characteristic curves (ROC), AUC (area under a curve), and Youden’s index were also used in the analysis. Statistica 12.0 software (StatSoft, Tulsa, OK, USA) was used for statistical analysis. The value of *p* < 0.05 was considered as the significance level.

A retrospective study was carried out in accordance with the rules of the Bioethical Committee of the Medical University of Warsaw.

## 3. Results

The results of laboratory and clinical analysis for subjects with κ and λ amyloid are presented in Table 1.

It has been proven that, with a decrease in the eGFR value the concentrations of NT-proBNP and the κ uninvolved chains increase significantly. The concentration of TnI, involved κ and λ FLC, uninvolved λ FLC, dFLC, and κ/λ values does not change notably (Table 2).

In the entire group of patients with myocardial involvement, a positive statistically significant correlation was demonstrated between the dFLC value and the concentrations of TnI and NT-proBNP, as well as between the concentration of the involved λ chains and the concentrations of TnI and NT-proBNP. There was a significant correlation between the concentrations of the involved κ and λ FLC, and the dFLC value. A negative statistically significant correlation was found between the eGFR value and the concentrations of NT-proBNP, uninvolved κ FLC, and the dFLC value, as well as between the κ/λ ratio and the concentrations of TnI, NT-proBNP, and the dFLC value. The highest significant correlations between dFLC values and cardiac parameters (TnI and NT-proBNP) were found in the group with eGFR ≥ 60 mL/min/1.73 m^2^; in the group with eGFR < 60 mL/min/1.73 m^2^, a similar correlation was not demonstrated. Similarly, in the case of the κ/λ values, negative statistically significant correlations with the TnI concentration and the dFLC value were confirmed in the entire group of patients, and with eGFR ≥ 60 mL/min/1.73 m^2^. The reduction of the eGFR value < 60 mL/min/1.73 m^2^ also resulted in the deterioration of the correlation between the concentration of the involved κ and λ FLC, and the dFLC value (Table 3).

In order to assess the diagnostic power of laboratory parameters used in the diagnosis of myocardial involvement in patients with AL amyloidosis in different eGFR groups, an ROC analysis was performed. The highest values of AUC were obtained for the NT-proBNP concentration, both in the whole study group, and for the eGFR ≥ 60 and < 60 mL/min/1.73 m^2^. The lowest values of the AUC and Youden’s index were obtained for the dFLC values, involved κ FLC concentration, and the κ/λ value for the calculated cut off >92.67 mg/L. For all compared parameters, the smallest values of the AUC were obtained for eGFR < 60 mL/min/1.73 m^2^. A decrease in eGFR value < 60 mL/min/1.73 m^2^ influenced the change in the cut-off points of all analyzed laboratory parameters. 

Over the entire study group, the highest sensitivity in relation to the diagnostics of myocardial involvement was demonstrated by involved λ FLC (91%) and NT-proBNP (89%) concentration, and the lowest by involved κ FLC (50%), TnI (61%) concentration, and the κ/λ value for the calculated cut off of >29.30 mg/L (2%) and <0.41 mg/L (60%). In turn, the highest specificity in the diagnosis of heart involvement by amyloid was obtained for the concentration of TnI (100%), and the κ/λ ratio for the determined cut-off values of >92.67 mg/L (100%) and <0.41 mg/L (89%) (Table 4).

## 4. Discussion

Authors of numerous studies have repeatedly pointed to the fact that both NT-proBNP and TnI concentrations, as well as sFLC concentrations, dFLC value, and κ/λ concentration ratio, depend on the kidney function and the eGFR values [1,10,18,19,20,21]. At the same time, it is pointed out that there is a necessity to perform more detailed analyses assessing the influence of renal dysfunction on the usefulness and diagnostic power of parameters used in the currently applicable prognostic classification of AL amyloidosis. Moreover, since cardiac involvement is the major adverse factor in AL amyloidosis, it is important to evaluate which laboratory parameters are most powerful in detecting the presence of amyloid deposits in the myocardium [8,22]. 

In the study group, both the concentrations of cardiac parameters TnI and NT-proBNP, as well as dFLC values and the percentage of plasmacytes in the bone marrow biopsy study, showed significantly higher values in patients with the presence of the λ chain, compared to patients with the monoclonal κ chain. Additionally, a significant correlation was confirmed between the concentration of the involved λ chains and the concentration of NT-proBNP, TnI, and the dFLC value. The described relationship has not been demonstrated in patients with the presence of monoclonal κ chains. These results may indicate that the presence of a monoclonal λ chain in patients with AL amyloidosis is associated with an increased risk of heart involvement, and thus a worse prognosis. 

Similar relationships were not demonstrated regarding eGFR values in the study group: no significant differences in glomerular filtration were observed in patients with κ and λ monoclonal hypertrophy. However, our own studies have shown that the eGFR value may affect the concentrations of laboratory parameters used in the prognostic classification of AL amyloidosis, including cardiac parameters, as well as the dFLC value and κ/λ index, which is a kind of numerical determinant of, among other factors, the clonality of plasmacytes in the bone marrow.

An important aspect is the influence of kidney function on the concentrations of NT-proBNP and TnI. While in the studied group the concentrations of chains involved in the neoplastic process did not change with the decrease in eGFR, the concentration of NT-proBNP was significantly increased in the group with eGFR < 15 mL/min/1.73 m^2^, and in the group with 15≥ eGFR < 60 mL/min/1.73 m^2^, compared to the group of eGFR ≥ 60 mL/min/1.73 m^2^. The results of the study confirmed that, as the eGFR value decreases, the concentration of NT-proBNP increases significantly. It is worth noting that this increase is independent of the concentration of the amyloidogenic chains of κ or λ, as there is no correlation between the deteriorating filtration function of the kidneys and the concentration of chains involved in the neoplastic process. It seems that the cardiac parameter used in the prognostic classification of AL amyloidosis, independent of renal function, is TnI—its concentration does not change as eGFR decreases. The above conclusions are also confirmed by the analysis of the correlation coefficients between the tested laboratory parameters; a statistically significant relationship was confirmed between the eGFR values and the concentration of NT-proBNP, but no similar correlation was demonstrated between the eGFR value and the TnI concentration (*p* > 0.05).

Interesting relationships were also found between the concentration of κ and λ FLC, the calculated indices dFLC and κ/λ, and the value of eGFR and cardiac parameters. Significant correlations between the dFLC and TnI, as well as between dFLC and NT-proBNP, were demonstrated in the entire study group, and in patients with eGFR ≥ 60 mL/min/1.73 m^2^; in contrast, similar relationships were not found in the group of eGFR < 60 mL/min/1.73. Significant correlations were also found between the concentration of the involved κ or λ chains and the dFLC value, which, notably, was the lowest in the group of patients with eGFR < 15mL/min/1.73 m^2^. This low dFLC value is a consequence of a significantly increasing concentration of polyclonal chains. In the study group, along with the deterioration of renal function, the concentration of uninvolved κ chains increased significantly, while, at the same time, the concentrations of the non-involved chains remained unchanged. A similar relationship was observed with regard to the correlation between the value of the κ/λ ratio and the concentrations of the κ and λ chains. With the decrease in eGFR, the value of the coefficient of negative correlation between the κ/λ ratio and the concentration of the λ involved chains decreased. With the decrease in eGFR, the value of the coefficient of positive correlation between the κ/λ ratio and the concentration of the κ involved chains decreased.

The relationships indicate that, in patients with impaired kidney function and a decrease in eGFR, the concentration of polyclonal light chains increases. This is due to the twice as large production of κ chains, and the simultaneous slowdown in the metabolism of both types of light chains in the renal tubules [23,24,25]. The dependence of the concentration of polyclonal sFLC on eGFR may therefore limit the usefulness of both the dFLC value and the κ/λ ratio in the prognostic classification of AL amyloidosis, and in the assessment of the myocardial function and damage degree, since the concentrations of both the chains involved in the neoplastic process and the uninvolved polyclonal chains are used for the calculation of the aforementioned parameters.

In our previous study, we showed that in the group of patients with AL amyloidosis with myocardial involvement, the only parameter to have an independent effect on the concentration of TnI was the concentration of λ chains involved. On the other hand, an independent effect on the concentration of NT-proBNP was demonstrated not only by the concentration of monoclonal λ chains, but also by the eGFR value, and, interestingly, by the concentration of polyclonal chains [26]. It can therefore be concluded that the biochemical parameters of the assessment of the myocardial function and damage degree in patients with AL amyloidosis depend not only on the concentration of monoclonal involved sFLC, but also on the function of kidney and eGFR value; this is especially applicable for the NT-proBNP concentrations. Numerous studies have shown that an increase in the concentration of polyclonal sFLCs is associated with an increased risk of cardiac death, both in patients with diagnosed diabetes and CKD, as well as in the general population [24,27,28,29,30]. So far, no similar studies have been performed in patients with AL amyloidosis.

In this study, an attempt was also made to select the laboratory parameter with the highest diagnostic power in the assessment of myocardial involvement in patients with AL amyloidosis and chronic kidney disease. In a comparative analysis of the parameters used in the classification of Kumar et al. [4], and additionally considered concentrations of the involved chains and the κ/λ ratio, our study confirmed that concentration of NT-proBNP has the largest diagnostic utility in the assessment of myocardial involvement, both in total and the eGFR ≥ 60, as well as the eGFR< 60 mL/min/1.73 m^2^ study groups. Notably, concentration of involved λ chains showed a high diagnostic power in the assessment of the heart involvement, comparable to the routinely used “cardiac parameters” TnI and NT-proBNP. It can therefore be an additional parameter used to assess the presence of amyloid in the myocardium.

The dFLC value, the concentration of the involved κ FLC, and the κ/λ value showed a very low diagnostic power in assessment of heart involvement, especially at eGFR < 60 mL/min/1.73 m^2^, which consequently disqualifies the use of these parameters in the assessment of the state of myocardial function in AL amyloidosis. It is worth noting, however, that the decrease in eGFR < 60 mL/min/1.73 m^2^ reduced the diagnostic power of all analyzed parameters, and changed the suggested cut-off points for discrimination between normal and abnormal values. Moreover, the decrease in eGFR resulted in a sensitivity decrease in the majority of the analyzed parameters.

The impact of kidney dysfunction on the usefulness of NT-proBNP concentration in the assessment of cardiac function in patients with AL amyloidosis was analyzed in the study by Palladini et al., where the authors have unequivocally stated that, so far, this problem was somewhat overlooked and downplayed. Palladini et al. have shown that the concentrations of natriuretic peptides (BNP and NT-proBNP) in patients with primary amyloidosis depend on both cardiac and kidney functions, especially regarding the concentration of NT-proBNP. The authors of the discussed study also indicate that with eGFR values < 15 mL/min/1.73 m^2^, the BNP concentration, unlike NT-proBNP, is an independent prognostic factor of survival, and therefore it should be a parameter used in the assessment of cardiac function in patients with end-stage renal disease [8]. In turn, the studies conducted by Palladini et al. in 2016 showed that the concentrations of NT-proBNP and TnI, as well as the value of dFLC, are all independent prognostic factors in AL amyloidosis, but only in the case of patients with preserved kidney function. In patients with eGFR < 30 mL/min/1.73 m^2^, however, only the concentration of TnI is an independent prognostic parameter, which was also confirmed in the present study. Moreover, in the studies of Palladini et al., it was proved that a decrease in eGFR reduces the sensitivity of the κ/λ index in the assessment of FLC clonality; however, the statistical significance of these differences was demonstrated only for monoclonal λ chains (81% vs. 60%; *p* < 0.001) [22]. On the other hand, in the publication by Halushka et al., it was indicated that only the value of the κ/λ concentration ratio in the range of 0.5–5.0 allows with 100% sensitivity and 100% specificity for the recognition of cases of AL amyloidosis among patients with other types of cardiac amyloidosis (transthyretin amyloidosis–ATTR and secondary AA amyloidosis) [31]. In the studies conducted by Capellie et al., in a group of 76 patients diagnosed with primary amyloidosis, it was clearly demonstrated that the TnI concentration showed a significantly higher sensitivity, specificity, and diagnostic power in the diagnosis of right ventricular dysfunction, compared to the concentration of NT-pro BNP. Simultaneously, it was suggested that NT-pro BNP is characterized by a greater dependence on kidney function, which may be the reason for a decrease in the diagnostic value of this parameter in the assessment of heart function in the analyzed group of patients [32].

The influence of kidney function on the concentration of laboratory parameters included in the prognostic classification and assessment of the myocardium condition in patients with AL amyloidosis is an important issue. It is predominant, since renal involvement in this group of patients occurs in approx. 2/3 of patients, and kidney failure is diagnosed in approximately 2/5 [33]. However, the described problem is not only a “laboratory dilemma”, where the dependence of NT-pro BNP and sFLC concentrations on the kidney function and tubular metabolism, as well as the influence of uremic toxins on the increase in troponin levels (in a mechanism yet to be fully discovered) are known, but it is also a clinical dilemma. The interdependence of heart and kidney functions in the case of patients with AL amyloidosis may resemble the so-called cardiorenal syndrome, which is defined as a group of diseases leading to pathophysiological disorders in the heart and kidneys, where a chronic or acute dysfunction of one of them leads to chronic or acute dysfunction of the other. The coexistence of heart and kidney failure often prevents the diagnosis of the primary cause (nephrological vs. cardiological) [34,35]. In the case of patients with AL amyloidosis, this mutual dependency may take the form of chronic cardiorenal syndrome (type 2) or chronic renocardiac syndrome (type 4). In such cases, cardiac dysfunction not only results from the presence of deposited amyloid or its precursors, but also shows a close relationship with kidney function and the eGFR value in a separate mechanism.

In conclusion, TnI appears to be the most relevant cardiac parameter used in the prognostic classification of AL amyloidosis, independent of renal function. Contrary to the NT-proBNP concentration, this parameter did not correlate with the glomerular filtration rate. Deterioration of renal function reduces NT-proBNP’s ability to reflect changes in myocardial function in patients with AL amyloidosis. Consequently, it somehow disqualifies this parameter from being used in prognostic classification and assessment of myocardial involvement by amyloid. From a practical point of view, it seems advisable to use only the concentration of TnI in the prognosis of the course of the disease in patients with decreased eGFR value. NT-proBNP can be used as a prognostic marker, but with different higher cut-off points in patients with decreased glomerular filtration. In our study, the proposed cut-off in the group with eGFR < 60 mL/min/1.73 m^2^ is the concentration of NT-proBNP > 4546 pg/mL. The dFLC value included in the current classification of Kumar et al. also seems to be a parameter of little use in patients with impaired renal function. The accumulation in the blood of uninvolved FLC, which is accompanied by the deterioration of the glomerular filtration, results in obtaining dFLC values which do not fully reflect the concentration of amyloidogenic monoclonal FLC present in the blood. A very low AUC surface area showed a dFLC value at eGFR < 60 mL/min/1.73 m^2^–only 0.550, which indicates the uselessness of this parameter in the assessment of the myocardial status in AL amyloidosis in patients with impaired renal filtration function. It is noteworthy that the concentration of λ chains involved had a higher diagnostic power in assessing cardiac involvement, compared to the routinely used TnI and NT-proBNP “cardiac parameters”. Thus, it may be an additional parameter for the assessment of the presence of amyloid in the myocardium, and thus also included in the prognostic classification of AL amyloidosis. The presence of the monoclonal λ chain, already at the initial stage of diagnosis, could indicate that the patient exhibits an increased risk of myocardial involvement by amyloid, which is inevitably associated with a poorer prognosis, and shorter overall survival. In our study, the concentration of the monoclonal λ chain above 68.10 mg/L with 91% sensitivity indicated the presence of amyloid in the heart. The decrease in the eGFR value changed the diagnostic cut-off points of the most frequently analyzed laboratory parameters, which should be taken into account in all patients diagnosed with primary amyloidosis who simultaneously experience a deterioration of the filtration function of the kidneys, irrespective of its causes.

## Figures and Tables

**Table 1:** Laboratory and clinical findings from enrolled patients.

Involved Chain	All Me (Q25–75)	Λ Me (Q25–75)	Κ Me (Q25–75)	*p*-Value
N	71	43	28	
Age (years)	58 (49–67)	59 (49–68)	56 (49–67)	0.661
Plasmacytes (%)	14 (6–19)	15 (10–20)	8 (6–15)	0.048
TnI (ng/mL)	0.040 (0.009–0.100)	0.065 (0.019–0.165)	0.022 (0.000–0.050)	0.033
NTproBNP(pg/mL)	2150 (400–6804)	4721 (1186–11,524)	432 (270–1810)	<0.001
κ (mg/L)	18.1 (12.4–51.9)	14.7 (10.9–19.3)	45.1 (21.3–140.3)	<0.001
λ (mg/L)	68.1 (16.5–215.3)	161.8 (88.3–312.8)	15.8 (12.6–28.3)	<0.001
dFLC (mg/L)	92.5 (7.7–236.9)	133.3 (29.2–298.7)	23.9 (5.8–109.7)	0.037
κ/λ	0.60 (0.10–1.69)	0.12 (0.04–0.50)	2.20 (1.38–5.14)	<0.001
eGFR (mL/min/1.73 m^2^)	71 (41–94)	74 (42–100)	69 (27–94)	0.430

TnI: troponin I; NT pro-BNP: N-terminal pro b-type natriuretic peptide; dFLC: difference between the concentration of involved and uninvolved free light chain; eGFR: estimated glomerular filtration rate.

**Table 2:** The comparative analysis of the study group depending on the stage of chronic kidney disease (CKD) according to Kidney Disease: Improving Global Outcomes (KDIGO) CKD Work Group: KDIGO 2012.

Group by eGFR [mL/min/1.73 m^2^]	≥60 (A) Me (Q25–75)	≥15<60 (B) Me (Q25–75)	<15 (C) Me (Q25–75)	*p*-Value
Age (years)	56 (47–65)	59 (53–63)	67 (57–73)	0.146
TnI (ng/mL)	0.040 (0.009–0.070)	0.120 (0.001–0.560)	0.035 (0.009–0.080)	0.450
NT–proBNP (pg/mL)	1323 (270–4780)	2340 (531–12,100)	8155 (1319–26,396)	0.037
κ–„i”(mg/L)	42.5 (13.6–85.1)	110.8 (28.2–463.7)	39.4 (27.4–193.0)	0.505
λ–„ui” (mg/L)	16.16 (9.0–23.6)	21.81 (14.0–47.2)	14.1 (3.6–46.4)	0.413
κ–„ui” (mg/L)	13.6 (10.8–16.3)	17.1 (10.6–27.4)	83.3 (53.4–121.3)	0.005
λ–„i” (mg/L)	188.7 (99.2–327.5)	197.1 (63.8–473.0)	114.8 (110.6–143.7)	0.670
dFLC (mg/L)	99.55 (12.8–285.49)	108.20 (6.46–462.40)	13.26 (−7.00–110.60)	0.198
κ/λ	0.22 (0.08–1.00)	1.30 (0.05–2.22)	0.98 (0.41–1.94)	0.244
eGFR (mL/min/1.73 m^2^)	92 (79–110)	42 (29–51)	13 (11–14)	<0.001

„i”: involved free chain; „ui”: uninvolved free chain; TnI: troponin I; NT pro-BNP: N-terminal pro b-type natriuretic peptide; dFLC: difference between the concentration of involved and uninvolved free light chain; eGFR: estimated glomerular filtration rate.

**Table 3:** Correlations of the concentrations of κ and λ free light chains (involved and uninvolved), dFLC values, and the κ/λ ratio with biochemical parameters of cardiac involvement and the eGFR values in the group with myocardial amyloid involvement, depending on the value of eGFR.

		TnI	NT-proBNP	eGFR	dFLC	κ/λ
eGFR	entire group	−0.180	−0.348 *	NA	0.133	−0.217
	eGFR ≥ 60 mL/min/1.73 m^2^	−0.300	−0.220	NA	0.091	−0.073
	eGFR < 60 mL/min/1.73 m^2^	0.251	−0.113	NA	0.145	−0.171
dFLC	entire group	0.416 *	0.364 *	0.133	NA	−0.441 *
	eGFR ≥ 60 mL/min/1.73 m^2^	0.559 *	0.648*	0.091	NA	−0.586
	GFR <60 mL/min/1.73 m^2^	0.310	0.115	0.145	NA	−0.210
κ/λ	entire group	−0.442 *	−0.464 *	−0.217	−0.441 *	NA
	eGFR ≥ 60 mL/min/1.73 m^2^	−0.536 *	−0.573 *	−0.073	−0.586 *	NA
	eGFR < 60 mL/min/1.73 m^2^	−0.365	−0.613 *	−0.171	−0.210	NA
κ–„i”	entire group	0.218	0.251	−0.158	0.932 *	0.848 *
	eGFR ≥ 60 mL/min/1.73 m^2^	0.219	0.434	0.227	0.921 *	0.878 *
	eGFR < 60 mL/min/1.73 m^2^	−0.371	−0.321	−0.146	0.891 *	0.800 *
λ–„ui”	entire group	0.188	0.253	−0.145	0.058	−0.219
	eGFR ≥ 60 mL/min/1.73 m^2^	0.188	0.028	−0.103	−0.121	−0.306
	eGFR < 60 mL/min/1.73 m^2^	0.314	0.455	0.191	0.109	−0.146
κ–„ui”	entire group	0.001	0.233	−0.400 ^#^	−0.419 ^#^	0.522*
	eGFR ≥ 60 mL/min/1.73 m^2^	−0.033	−0.023	−0.012	−0.336	0.366
	eGFR < 60 mL/min/1.73 m^2^	0.063	0.464	−0.582	−0.439	0.546
Λ–„i”	entire group	0.527 *	0.445 ^#^	0.110	0.965 *	−0.912 *
	eGFR ≥ 60 mL/min/1.73 m^2^	0.410	0.438	0.035	0.997 *	−0.954 *
	eGFR < 60 mL/min/1.73 m^2^	0.725 *	0.622	0.193	0.921 *	−0.879 *

* *p* < 0.005, ^#^ *p* < 0.01; NA: not applicable; „i”: involved free chain; „ui”: uninvolved free chain; TnI: troponin I; NT pro-BNP: N-terminal pro b-type natriuretic peptide; dFLC:difference between the concentration of involved and uninvolved free light chain; eGFR: estimated glomerular filtration rate.

**Table 4:** Results of the ROC curves analysis for the concentration of TnI, NT-proBNP, and dFLC values in the diagnostics of myocardial involvement depending on the eGFR value.

	eGFR	AUC	Youden’s Index	Cut Off	Sensitivity [%]	Specificity [%]
TnI	entire group	0.823	0.61	0.05	61	100
	eGFR ≥ 60 mL/min/1.73 m^2^	0.883	0.66	0.009	96	70
	eGFR < 60 mL/min/1.73 m^2^	0.713	0.67	0.08	67	100
NT-pro BNP	entire group	0.906	0.68	500	89	79
	eGFR ≥ 60 mL/min/1.73 m^2^	0.914	0.69	973	84	85
	GFR <60 mL/min/1.73 m^2^	0.881	0.67	4546	67	100
dFLC	entire group	0.723	0.47	45.40	75	72
	eGFR ≥ 60 mL/min/1.73 m^2^	0.855	0.64	23.86	89	75
	eGFR < 60 mL/min/1.73 m^2^	0.550	0.32	45.40	65	67
κ–„i”	entire group	0.630	0.32	85.07	50	82
	eGFR ≥ 60 mL/min/1.73 m^2^	0.792	0.58	45.06	75	83
	eGFR < 60 mL/min/1.73 m^2^	0.400	0.10	138.00	50	60
λ–„i”	entire group	0.840	0.62	68.10	91	71
	eGFR ≥ 60 mL/min/1.73 m^2^	0.886	0.68	188.66	68	100
	eGFR < 60 mL/min/1.73 m^2^	0.857	0.86	68.10	86	100
κ/λ	entire group	0.733	0.48	<0.41	60	89
	eGFR ≥ 60 mL/min/1.73 m^2^	0.715	0.58	<0.2	67	92
	eGFR < 60 mL/min/1.73 m^2^	0.9	0.8	<1.38	80	100

„i”: involved free chain; TnI: troponin I; NT pro-BNP: N-terminal pro b-type natriuretic peptide; dFLC: difference between the concentration of involved and uninvolved free light chain; eGFR: estimated glomerular filtration rate, AUC–area under curve.

## Data Availability

The data presented in this study are available on request from the corresponding author. The data are not publicly available due to ethical reasons.
